# The role of tissue IgG4 levels in steroid therapy in patients with idiopathic granulomatous mastitis

**DOI:** 10.1007/s10238-024-01444-7

**Published:** 2024-07-28

**Authors:** Celil Seyidli, Yunushan Furkan Aydoğdu, Çağrı Büyükkasap, Ramazan Kozan, Mahir Nasirov, Kürşat Dikmen, Güldal Esendağli Yilmaz, Murat Akin

**Affiliations:** 1 Department of General Surgery, Kocaeli Atakent Cihan Hospital, İzmit, Kocaeli Turkey; 2 Department of General Surgery, Bandırma Research and Training Hospital, Balikesir, Turkey; 3 Department of General Surgery, Faculty of Medicine, Gazi University, Ankara, Turkey; 4 Caspian International Hospital, Baku, Azerbaijan; 5 Department of Medical Pathology, Faculty of Medicine, Gazi University, Ankara, Turkey

**Keywords:** Immunoglobulin G, IgG4, Idiopathic granulomatous mastitis, Steroid therapy, Recurrence

## Abstract

Idiopathic granulomatous mastitis (IGM) is a benign, chronic inflammatory lesion of the breast. Immunoglobulin G4 (IgG4) associated disease is rare in the breast. In our study, we aimed to evaluate the efficacy of steroid treatment on IgG4 levels in tissue in patients diagnosed with IGM. Between 2008 and 2017, 55 patients diagnosed with IGM in our clinic were included in the study. Demographic, clinical, microbiologic and histopathologic characteristics, treatment modality and recovery time were evaluated retrospectively. Patients were divided into 3 groups according to tissue IgG4 levels: negative (Group I), infrequently and slightly positive (Group II), and highly positive (Group III). Group I patients had a complete response rate of 77.8%. In the rest of the patients (22.2%), insufficient response was detected from the beginning of the treatment. In Group II, the response rate was 91.3% and the permanent success rate after treatment was 87.0%. Although group III patients had a complete response at the beginning (95.65%), they relapsed in a short period of time (26.1%) after discontinuation of steroid treatment. At least one steroid-related side effect was observed in 47 (85.8%) patients in all groups. There is no consensus on the dose and duration of immunosuppressive treatment in IGM. In this study, responses to steroid treatment according to IgG4 concentration in pathologic breast tissue and recurrences after the end of treatment were determined. We think that high IgG4 concentration in the tissue is associated with recurrence and other immunosuppressive drugs should be added as maintenance after steroid treatment.

## Introduction

The term mastitis generally describes the inflammation of part or all of the breast tissue for various reasons. The breast is an organ vulnerable to inflammatory events because it is anatomically located on the outer surface of the body, it is an excellent medium for microorganisms during milk secretion and it is exposed to constant trauma during breastfeeding. Inflammatory lesions of the breast are frequently encountered between the ages of 18 and 50 years, but are not usual except in the postpartum period [1–3]. Inflammation in breast tissue can mimic the clinical manifestations of many diseases ranging from simple mastitis to inflammatory breast cancer. A detailed patient history and some clinical features help the diagnosis [4].

Granulomatous mastitis (GM) is a rare, benign, chronic inflammatory lesion of the breast of unknown etiology [5, 6]. The etiology of IGM has not been fully elucidated. In the literature, good response to steroid and immunosuppressive treatments, good response to steroid treatment in patients with recurrence after surgical treatment, reports of patients with extra- breast involvement such as erythema nodosum or arthritis, and T-lymphocyte predominance in immunohistochemical studies are reported as findings supporting the autoimmunity hypothesis [7].

IGM has been reported to be unrelated to pregnancy status, with an increased number of IgG4 positive plasma cells. In addition to this morphologic spectrum of lesions, IgG4-associated lesions have been observed. Assuming that IgG4-positive plasma cells are involved in the pathogenesis of other plasma cell-rich lesions in the breast, plasma cell mastitis, idiopathic granulomatous mastitis, non-specific mastitis, inflammatory pseudotumor and other plasma cell-rich, mass-forming benign inflammatory lesions have been investigated as possible members of IgG4-related sclerosing mastitis [8–10].

IgG4-related mastitis is used to describe IgG4-related disease of the breast. Like all other IgG4-associated diseases, it is clinically relevant as it can mimic malignant conditions and coexist with other systemic manifestations. In contrast to many cases in other organs, very few IgG4-associated diseases have been reported so far in the breast. In general, there are contradictions in the diagnosis and treatment of patients diagnosed with IGM and recurrence rates are high. Therefore, in our study, we aimed to evaluate the efficacy of steroid treatment according to IgG4 level in tissue in patients diagnosed with IGM.

## Materials and methods

Between January 2008 and June 2017, 74 patients diagnosed with IGM in our clinic who were started on methyl-prednisolone 1 mg/kg/day in the first week and the dose was reduced to 0.1 mg/kg/day per week were evaluated. Patients were evaluated retrospectively according to pregnancy, breastfeeding, smoking, oral contraceptive (OCS) use, clinical findings, diagnostic tests, microbiologic and histopathologic features and treatment modality.

Inclusion criteria (n = 74); histopathologically diagnosed as GM, receiving steroid treatment after diagnosis (Methyl prednisolone), patients > 18 years of age, patients without surgical excision, patients diagnosed by tru-cut biopsy, it was determined as fixation of all specimens with 10% formaldehyde.

Exclusion criteria (n = 19) were as follows: patients with incomplete records, patients with a follow-up period of less than 3 months, patients who did not comply with the follow-up and treatment protocol, patients diagnosed with breast cancer before or after treatment, patients who underwent surgery, patients who received chemotherapy and/or radiotherapy to the thorax for any reason, pregnant patients, patients receiving immunosuppressive treatment other than steroids.

After excluding patients who did not meet the mentioned criteria, a total of 55 patients were studied. The specimens and slides of these patients, which were fixed with 10% formaldehyde and embedded in paraffin, were analyzed and appropriate blocks were selected and prepared for staining.

### Immunohistochemical method

Paraffin blocks containing tissue samples fixed with 10% formaldehyde solution and selected to best represent the lesion from H&E sections, 4 µm thick sections were prepared on positively charged slides with biopsy numbers indicated, with two cases on one slide. In order to determine the expression of Cell Marque Marker IgG4 (MRQ-44) Mouse Monoclonal Primary Antibody in these sections, automatic immunohistochemistry staining was performed on Ventana Benchmark XT closed device. Tonsil tissue was used as a positive control.

The steps of the immunohistochemical staining method applied are listed below;

- 1. Tissue sections 4 mm thick were taken on positively charged slides.
- 2. Slides were placed on a Ventana^®^ (Roche, Switzerland).
- 3. IgG4 was placed in EDTA buffer (Ph:8.0) for 30 min.
- 4. For primary antibody incubation, IgG4 was kept in the device for 32 min.
- 5. Ultraview Universal Alkaline Phosphatase Red Detection Kit^®^ (Roche, Switzerland); was used for colorimetric imaging.
- 6. Contrast staining was completed with Ventana brand hematoxylin I.
- 7. The slides were washed in tap water and kept in alcohol and xylol for 2 min, respectively.
- 8. Slides were sealed using Entellan^®^ (Merck, Germany).

After the procedures, the patients were analyzed in three groups according to the IgG4 level in the tissue.

**Fig. 1:**
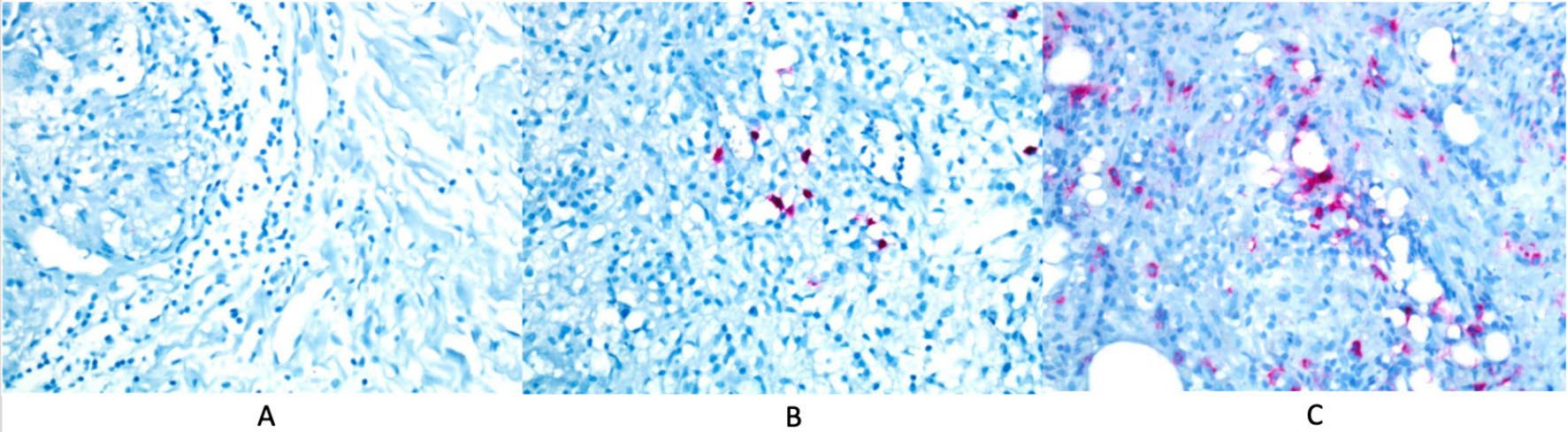
**A** Tissue IgG4 negative (absence of IgG4 positive staining plasma cells in microscopic 10 large magnification field, ×400). **B** IgG4 in the tissue was sparse and few (less than 10 IgG4 positively stained plasma cells in 10 microscopic fields of magnification, ×400). **C** IgG4 in the tissue was localized and numerous (microscopic 10 large magnification field with 11 or more IgG4 positively stained plasma cells, ×400)

*Group I*: 9 patients (16.3%). Tissue IgG4 negative (absence of IgG4 positive staining plasma cells in microscopic 10 large magnification field, ×400) (Fig. 1A).

*Group II*: 23 patients (41.8%). IgG4 in the tissue was sparse and few (less than 10 IgG4 positively stained plasma cells in 10 microscopic fields of magnification, ×400) (Fig. 1B).

*Group III*: 23 patients (41.8%). IgG4 in the tissue was localized and numerous (microscopic 10 large magnification field with 11 or more IgG4 positively stained plasma cells, ×400) (Fig. 1C).

In these patients, additional parameters were analyzed by reviewing the patients; age, localization, time between last pregnancy and diagnosis, use of OCS, duration of steroid use, breastfeeding, cigarette smoking, additional disease, prolactin level, serum IgG level, acid-resistant bacilli (ARB) and fungal infections were not observed in the examination of tissue samples of all patients.

### Statistical analysis

All statistical analyses were performed with IBM SPSS version 26.0 (IBM Co, Armonk, NY, USA). Continuous data were analyzed using mean, median, standard deviation and 95% confidence intervals (CIs). Chi-square test was used for comparative analysis of categorical data. Mann Whitney *U* test was used for comparative analysis of non-categorical data. < 0.05 values were considered statistically significant. The ethics committee of the study was obtained from Gazi University Ethics Committee (23/10/2018-497).

## Results

The median age of the 55 patients included in the study was 35 years (min–max: 24–56). The mean prolactin level was 11.9 ± 6 ng/dl (pre-menopausal (< 50 years): 3.34 ± 26.7 ng/dl; postmenopausal (> 50 years): 2.74 ± 19.6 ng/dl), serum IgG level mean 1127 ± 341.8 mg/dl, median interval between pregnancy and diagnosis was 51 months (min–max: 5–240), median follow-up period was 24 months (min–max: 4–82), median duration of steroid use was 60 days (min–max: 30–360) (Table 1).

**Table 1:** Demographic characteristics, serum prolactin and serum IgG levels, follow-up periods and steroid duration

Age, median (range), year	35 (24–56)
Prolaktin, mean ± SD, ng/dl Pre-menopause Post menopause	11.9 ± 6 3.34 ± 26.7 2.74 ± 19.6
Serum IgG, mean ± SD, mg/dl	1127 ± 341.8
Interval between pregnancy and diagnosis, median (range), month	51 (5–240)
Follow-up time, median (range), month	24 (4–82)
Duration of steroid use, median (range), day	60 (30–360)

SD, Standard deviation; ng, nanograms; dl, deciliter; mg, milligrams

There was no significant difference between groups I, II and III in terms of age, prolactin levels, serum IgG4 level, duration of steroid use, interval between pregnancy, follow-up period, pregnancy (delivery) status, breastfeeding, localization, OCS and smoking, breast discharge, abscess and mass formation, but there was a statistically significant difference in the presence of redness and edema (*p* = 0.018) (Table 2).

**Table 2:** Comparison of clinical characteristics of the groups

	Group I	Group II	Group III	Total	*p* value
Age, median (range), year	32 (24–44)	34 (24–56)	34 (25–54)		0.774
Prolactin, Mean ± SD, ng/dl	12.2 ± 11.2	9.7 ± 3.5	14 ± 4.1		0.186
Serum IgG4, Mean ± SD, mg/dl	852.5 ± 137.7	1122.1 ± 266.8	1277.3 ± 427.5		0.144
Interval between pregnancy and diagnosis, median (range), month	41 (24–96)	53 (5–240)	54 (12–226)		0.912
Follow-up time, median (range), month	36 (4–82)	22 (6–72)	26 (7–72)		0.876
Duration of steroid use, median (range), day	60 (45–110)	60 (30–180)	60 (45–360)		0.514
Pregnancy, n (%)					
Positive	8 (88.9%)	20 (87.0%)	20 (87.0%)	48 (87.3%)	0.903
Negative	1 (11.1%)	3 (13.0%)	3 (13.0%)	7 (12.7%)	
Breastfeeding, n (%)					
Positive	7 (77.8%)	18 (78.3%)	18 (78.3%)	43 (78.2%)	0.980
Negative	2 (22.2%)	5 (21.7%)	5 (21.7%)	12 (21.8%)	
Localization, n (%)					
Right	2 (22.2%)	6 (26.1%)	11 (47.8%)	19 (34.5%)	0.105
Left	7 (77.8%)	16 (69.6%)	12 (52.2%)	35 (63.6%)	
Bilateral	–	1 (4.3%)	–	1 (1.8%)	
OCS use, n (%)					
Positive	1 (11.1%)	3 (13.0%)	3 (13.0%)	7 (12.7%)	0.903
Negative	8 (88.9%)	20 (87.0%)	20 (87.0%)	48 (87.3%)	
Smoking, n (%)					
Positive	4 (44.4%)	5 (21.7%)	5 (21.7%)	14 (25.5%)	0.274
Negative	5 (55.6%)	18 (78.3%)	18 (78.3%)	41 (74.5%)	
Comorbidity, n (%)					
Positive	–	2 (8.7%)	3 (13.0%)	5 (9.1%)	0.264
Negative	9 (100.0%)	21 (91.3%)	20 (87.0%)	50 (90.9%)	
Breast discharge, n (%)					
Positive	5 (55.6%)	11 (47.8%)	8 (34.8%)	24 (43.6%)	0.244
Negative	4 (44.4%)	12 (52.2%)	15 (65.2%)	31 (56.4%)	
Redness and Edema, n (%)					
Positive	9 (100.0%)	15 (65.2%)	10 (43.5%)	34 (61.8%)	**0.018**
Negative	–	8 (34.8%)	13 (56.5%)	21 (38.2%)	
Abscess, n (%)					
Positive	8 (88.9%)	14 (60.9%)	14 (60.9%)	36 (65.5%)	0.216
Negative	1 (11.1%)	9 (39.1%)	9 (39.1%)	19 (34.5%)	
Palpable mass, n (%)					
Positive	5 (55.6%)	17 (73.9%)	18 (78.3%)	40 (72.7%)	0.240
Negative	4 (44.4%)	6 (26.1%)	5 (21.7%)	15 (27.3%)	

Bold values indicate statistically significant *p* values (*p* < 0.05)

SD, Standard deviation; ng, nanograms; dl, deciliter; mg, milligrams; IgG4, Immunoglobulin G4; OCS, Oral contraceptive; Group I, Tissue IgG4 negative (absence of IgG4 positive staining plasma cells in microscopic 10 large magnification field, ×400); Group II, IgG4 in the tissue was sparse and few (less than 10 IgG4 positively stained plasma cells in 10 microscopic fields of magnification, ×400); Group III, IgG4 in the tissue was localized and numerous (microscopic 10 large magnification field with 11 or more IgG4 positively stained plasma cells, ×400); n, number

A total of 11 (20.0%) patients, including 1 (11.1%) patient in Group I, 4 (17.4%) patients in Group II, and 6 (26.1%) patients in Group III, received steroid treatment again after treatment. Steroid-related side effects (hirsutism, weight gain, steroid acne, edema, hypertension, diabetes, hair loss, gastritis, cataract, hypothyroidism) were seen in 8 (88.9%) patients in Group I, 20 (87.0%) patients in Group II, 19 (82.6%) patients in Group III and 47 (85.8%) patients in total. Drainage procedure was performed in 4 (44.4%) patients in Group I, 12 (52.2%) patients in Group II, 15 (65.2%) patients in Group III and 31 (56.4%) patients in total. Recurrence was seen in 2 (22.2%) patients in Group I, 3 (13.0%) patients in Group II, 6 (26.1%) patients in Group III and 11 (20.0%) patients in total. There was no statistically significant difference between these parameters and the groups (Table 3).

**Table 3:** Treatment and recurrence results of patients diagnosed with GM according to patient groups

	Group I	Group II	Group III	Total	*p* value
Steroid additional dose, n (%)					
Positive	1 (11.1%)	4 (17.4%)	6 (26.1%)	11 (20.0%)	0.307
Negative	8 (88.9%)	19 (82.6%)	17 (73.9%)	44 (80.0%)	
Steroid complication, n (%)					
Positive	8 (88.9%)	20 (87.0%)	19 (82.6%)	47 (85.8%)	0.612
Negative	1 (11.1%)	3 (13.0%)	4 (17.4%)	8 (14.5%)	
Drainage, n (%)					
Positive	4 (44.4%)	12 (52.2%)	15 (65.2%)	31 (56.4%)	0.244
Negative	5 (55.6%)	11 (47.8%)	8 (34.8%)	24 (43.6%)	
Recurrence, n (%)					
Positive	2 (22.2%)	3 (13.0%)	6 (26.1%)	11 (20.0%)	0.577
Negative	7 (77.8%)	20 (87.0%)	17 (73.9%)	44 (80.0%)	

Group I, Tissue IgG4 negative (absence of IgG4 positive staining plasma cells in microscopic 10 large magnification field, ×400); Group II, IgG4 in the tissue was sparse and few (less than 10 IgG4 positively stained plasma cells in 10 microscopic fields of magnification, ×400); Group III: IgG4 in the tissue was localized and numerous (microscopic 10 large magnification field with 11 or more IgG4 positively stained plasma cells, ×400); n, number

Recurrence occurred in 6 (19.4%) of 31 patients who underwent drainage. Recurrence occurred in 1 (14.3%) of 7 patients using OCS. Recurrence occurred in 2 (14.3%) of 14 patients who smoked, and recurrence occurred in 10 (23.3%) of 43 patients with a history of breastfeeding. Recurrence occurred in 10 (20.8%) of 48 patients who had a history of pregnancy and gave birth. When these parameters were evaluated, no statistically significant difference was found. The median age of patients with recurrence was 24 years (min–max: 24–46) and the median age of patients without recurrence was 35 years (min–max: 24–56). The mean prolactin level was 17.3 ± 10.2 ng/dl in patients with recurrence and 10.8 ± 4.6 ng/dl in patients without recurrence. Serum IgG level was mean 974.0 ± 342.4 mg/dl in patients with recurrence and mean 1151.1 ± 342.8 mg/dl in patients without recurrence. The median time from the last pregnancy to diagnosis was 35 (min–max: 12–72) months in patients with recurrence and 60 (min–max: 5–240) months in patients without recurrence. Patients with recurrence used steroids for a median of 90 (min–max: 60–360) days, while patients without recurrence used steroids for a median of 60 (min–max: 30–180) days. The median follow-up period of patients with recurrence was 14 (min–max: 7–72) months, while the median follow- up period of patients without recurrence was 24 (min–max: 4–82) months. When these parameters were evaluated, the time between pregnancy and diagnosis (*p* = 0.027) and the duration of steroid use (*p* = 0.008) were statistically significant (Table 4).

**Table 4:** Evaluation of the effects of clinical characteristics on recurrence

	Recurrence		Total	*p* value
	Positive	Negative		
Drainage, n (%)				
Positive	6 (19.4%)	25 (80.6%)	31	0.577
Negative	5 (20.8%)	19 (79.2%)	24	
OCS use, n (%)				
Positive	1 (14.3%)	6 (85.7%)	7	0.571
Negative	10 (20.8%)	38 (79.2%)	48	
Smoking, n (%)				
Positive	2 (14.3%)	12 (85.7%)	14	0.424
Negative	9 (22.0%)	32 (78.0%)	41	
Breastfeeding, n (%)				
Positive	10 (23.3%)	33 (76.7%)	43	0.240
Negative	1 (8.3%)	11 (91.7%)	12	
Pregnancy, n (%)				
Positive	10 (20.8%)	38 (79.2%)	48	0.571
Negative	1 (14.3%)	6 (85.7%)	7	
Age, median (range), year	29 (24–46)	35(24–56)		0.075
Prolactin, Mean ± SD, ng/dl	17.3 ± 10.2	10.8 ± 4.6		0.233
Serum IgG4, Mean ± SD, mg/dl	974.0 ± 342.4	1151.1 ± 342.8		0.422
Interval between pregnancy and diagnosis, median (range), month	35 (12–72)	60 (5–240)		0.027
Duration of steroid use, median (range), day	90 (60–360)	60 (30–180)		0.008
Follow-up time, median (range), month	14 (7–72)	24 (4–82)		0.461

SD, Standard deviation; ng, nanograms; dl, deciliter; mg, milligrams; IgG4, Immunoglobulin G4; OCS, Oral contraceptive; Group I, Tissue IgG4 negative (absence of IgG4 positive staining plasma cells in microscopic 10 large magnification field, ×400); Group II, IgG4 in the tissue was sparse and few (less than 10 IgG4 positively stained plasma cells in 10 microscopic fields of magnification, ×400); Group III, IgG4 in the tissue was localized and numerous (microscopic 10 large magnification field with 11 or more IgG4 positively stained plasma cells, ×400); n, number

Weight gain due to steroid use was seen in 5 (55.6%) patients in Group I, 14 (60.9%) patients in Group II, 13 (56.5%) patients in Group III and 32 (58.2%) patients in total. Hirsutism was seen in 8 (88.9%) patients in Group I, 15 (65.2%) patients in Group II, 14 (60.9%) patients in Group III and 37 (67.2%) patients in total. Steroid acne was seen in 4 (44.4%) patients in Group I, 12 (52.2%) patients in Group II, 11 (47.8%) patients in Group III and 27 (49.1%) patients in total. There was no statistically significant difference between these parameters and the groups (Table 5).

**Table 5:** Distribution of side effects related to steroid use in patients according to groups

	Group I	Group II	Group III	Total	*p* value
Weight gain, n (%)					
Positive	5 (55.6%)	14 (60.9%)	13 (56.5%)	32 (58.2%)	0.942
Negative	4 (44.4%)	9 (39.1%)	10 (43.5%)	23 (41.8%)	
Hirsutism, n (%)					
Positive	8 (88.9%)	15 (65.2%)	14 (60.9%)	37 (67.2%)	0.304
Negative	1 (11.1%)	8 (34.8%)	9 (39.1%)	18 (32.8%)	
Acne, n (%)					
Positive	4 (44.4%)	12 (52.2%)	11 (47.8%)	27 (49.1%)	0.914
Negative	5 (55.6%)	11 (47.8%)	12 (52.2%)	28 (50.9%)	

Group I, Tissue IgG4 negative (absence of IgG4 positive staining plasma cells in microscopic 10 large magnification field, ×400); Group II, IgG4 in the tissue was sparse and few (less than 10 IgG4 positively stained plasma cells in 10 microscopic fields of magnification, ×400); Group III, IgG4 in the tissue was localized and numerous (microscopic 10 large magnification field with 11 or more IgG4 positively stained plasma cells, ×400); n, number

## Discussion

The number of cases of IGM is gradually increasing. Although there are many studies on clinical and radiologic findings and treatment methods, there is no established diagnostic and treatment protocol. IGM negatively affects the quality of life of patients because it is a difficult disease to manage. In the literature, the mean age of patients with IGM in recent studies is Houlihan E et al. [11], 45 years, Azzam MI et al. [12] 37 years, Lermi N et al. [13] 35 years, Esmaeil NK et al. [14] were evaluated as 35.7 years. In our study, the median age was 35 years. According to the literature and the results of our study, the incidence of IGM increases in the 3rd and 4th decade.

In our study, the relationship between the history of childbirth and breastfeeding and IgG4 level was not statistically significant, but the relationship between the time elapsed after pregnancy until diagnosis and recurrence was statistically significant. The fact that no significant difference was found between these factors and IgG4 level supported that breastfeeding and giving birth were not effective in the etiology.

Smoking in the etiology of IGM has not been clarified. Al-Khaffaf D et al. [15] and Altintoprak F et al. [16] reported that smoking was not effective, while Fattahi AS et al. [17] reported that smoking played a minimal role in the etiology. In our study, 14 (25.5%) patients (4 (44.4%) in Group I, 5 (21.7%) in Group II and 5 (21.7%) in Group III) were smokers. Although there was no significant difference between the groups, the fact that it was observed in 25.5% of the patients supported that it may be effective in the etiology. Studies have been conducted considering that hyperprolactinemia, like other hormone disorders, may be responsible for the pathogenesis based on the secretion theory [18, 19]. In our study, prolactin levels of the patients were evaluated and the groups were compared individually. A mean value of 12.2 ± 11.2 ng/dl was obtained in Group I, 9.7 ± 3.5 ng/dl in Group II and 14 ± 4.1 ng/dl in Group III. When evaluated according to the groups, prolactin level was not statistically significant.

The treatment of IGM is still unclear. Clinical observation and radiologic follow-up are especially important in suspected cases. Steroid treatment can be used preoperatively or postoperatively. There are proponents and non-proponents that steroid treatment is effective. Lermi N et al. [13] reported a 47.5% recurrence rate. Ren et al. [20] reported a recurrence rate of 17.7%. In our study, all patients were treated with methyl-prednisolone for a median of 60 (30–360) days, starting with 1 mg/kg/day in the first week and decreasing the dose to 0.1 mg/kg/day weekly. A total of 11 (20%) patients (1 (11.1%) in Group I, 4 (17.4%) in Group II, and 6 (26.1%) in Group III received additional steroid treatment. Recurrence was seen in 11 (20%) of 55 patients in our study. Recurrence was seen in 2 (22.2%) patients in Group I, 3 (13.0%) in Group II and 6 (26.1%) in Group III. There was no statistically significant difference between the recurrence and the groups. Steroid-related side effects were observed in 8 (88.9%) patients in Group I, 20 (87%) patients in Group II, 19 (82.6%) patients in Group III and 47 (85.8%) patients in total.

Since IgG4-related disease is a new concept worldwide, international diagnostic criteria are still being developed. The diagnostic criteria presented so far are based on two basic elements such as serum IgG4 elevation and histopathology. IgG4-associated disease can affect many organs together with the breast, and it is obvious that patients sometimes undergo unnecessary surgery because of the difficulties in the differential diagnosis from malignancy [8, 9, 21]. Although tissue IgG4 levels are usually found to be high, they are not sufficient for diagnosis. When we consider the breast, the role of IgG4 in the pathogenesis of the disease has not yet been fully established and the number of patients is limited in the literature [22, 23].

In our study, we evaluated the efficacy of steroid treatment in patients with high tissue IgG4 levels based on autoimmune etiology. Although our results showed no difference between the groups in the efficacy of steroid treatment, the treatment success rate was 77.8% in Group I, 87.0% in Group II and 73.9% in Group III. From this point of view, the response of steroid treatment in IgG4-associated granulomatous mastitis was compatible with the other groups in our study.

Our study has limitations, including the fact that factors such as the size of the swelling and concurrent treatment with immunomodulators like methotrexate or azathioprine were not considered. These factors can influence the recurrence rate of IGM and should be evaluated in future studies with larger and more detailed data collection. The variability in response to steroid therapy is well-documented in the literature, and the addition of adjunctive immunosuppressive treatments is a common practice. In our study, invasive IgG4 measurements were conducted to understand the relationship between IgG4 levels and steroid response, which can provide important insights for optimizing treatment plans. However, adjunctive immunosuppressive therapies, such as methotrexate and azathioprine, have a more favorable long-term side effect profile compared to steroids and should be considered to reduce recurrence rates and minimize side effects. Invasive IgG4 measurements are essential for understanding the pathogenesis of IgG4-related diseases and their response to steroid therapy. High IgG4 concentrations in tissue can provide critical information for tailoring patient-specific treatment plans. Our findings suggest that IgG4 levels are correlated with steroid response, highlighting the need for accurate and specific diagnostic tools to improve clinical outcomes.

## Conclusion

The etiology of idiopathic GM has not been fully elucidated. Autoimmunity is presumed to be involved in the etiology. The efficacy of immunosuppressive treatment also supports this. However, there is no consensus on the dose and duration of this treatment. In this study, we evaluated the responses to steroid treatment according to IgG4 concentration in pathologic breast tissue and recurrences after the end of treatment. Although the presence of IgG4 in the tissue (11 or more IgG4-positive plasma cells in 10 microscopic magnification fields) has the highest response to treatment, we think that other immunosuppressive drugs should be added as maintenance after steroid treatment in IgG4-positive mastitis due to the presence of recurrences in a short time and the side effects of steroid treatment. Our findings on the correlation between IgG4 levels and steroid response should be regarded as preliminary observations. These results need to be validated with larger, prospective studies to establish definitive conclusions and to develop standardized treatment protocols.

## Data Availability

The database of this study is open to sharing. It can be obtained from the authors upon request. Corresponding Author: Yunushan Furkan Aydoğdu. Department of General Surgery, Bandırma Training and Research Hospital Balıkesir/Turkey.

## Declarations

### Scientific responsibility statement

The authors declare that they are responsible for the scientific content of the article, including study design, data collection, analysis and interpretation, writing, part or all of the outline, preparation and scientific review of the content, and approval of the final version of the article.
